# Transient Prepubertal Mifepristone Treatment Normalizes Deficits in Contextual Memory and Neuronal Activity of Adult Male Rats Exposed to Maternal Deprivation

**DOI:** 10.1523/ENEURO.0253-17.2017

**Published:** 2017-11-02

**Authors:** Manila Loi, Ratna Angela Sarabdjitsingh, Andromachi Tsouli, Stephanie Trinh, Marit Arp, Harmen J. Krugers, Henk Karst, Ruud van den Bos, Marian Joëls

**Affiliations:** 1 Department of Translational Neuroscience, Brain Center Rudolf Magnus, UMC Utrecht, Utrecht, 3584 CG, The Netherlands; 2 Swammerdam Institute for Life Sciences-Center for Neuroscience, University of Amsterdam, Amsterdam, 1090 GE, The Netherlands; 3 Institute for Water and Wetland Research, Animal Ecology and Physiology, Faculty of Science, Radboud University, Nijmegen, Nijmegen, 6500 GL, The Netherlands; 4 University of Groningen/University Medical Center Groningen, Groningen, 9713 AV, The Netherlands

**Keywords:** early life stress, glutamate transmission, mifepristone, reward-based decision making, Rodent Iowa Gambling Task, spatial memory

## Abstract

Early life adversity is a well-known risk factor for behavioral dysfunction later in life, including the formation of contextual memory; it is also (transiently) accompanied by hyperactivity of the stress system. We tested whether mifepristone (MIF) treatment, which among other things blocks glucocorticoid receptors (GRs), during the prepubertal period [postnatal days (PND)26-PND28] normalizes memory deficits in adult male rats exposed to 24-h maternal deprivation (MD) at PND3. MD reduced body weight gain and increased basal corticosterone (CORT) levels during the PND26, but not in adulthood. In adulthood, contextual memory formation of MD compared to noMD (i.e., control) male rats was significantly impaired. This impairment was fully prevented by MIF treatment at PND26-PND28, whereas MIF by itself did not affect behavior. A second behavioral test, a rodent version of the Iowa Gambling Task (rIGT), revealed that flexible spatial learning rather than reward-based aspects of performance was impaired by MD; the deficit was prevented by MIF. Neuronal activity as tested by c-Fos staining in the latter task revealed changes in the right hippocampal-dorsomedial striatal pathway, but not in prefrontal areas involved in reward learning. Follow-up electrophysiological recordings measuring spontaneous glutamate transmission showed reduced frequency of miniature postsynaptic excitatory currents in adult CA1 dorsal hippocampal and enhanced frequency in dorsomedial striatal neurons from MD versus noMD rats, which was not seen in MIF-treated rats. We conclude that transient prepubertal MIF treatment normalizes hippocampus-striatal-dependent contextual memory/spatial learning deficits in male rats exposed to early life adversity, possibly by normalizing glutamatergic transmission.

## Significance Statement

We used a translational mouse model for severe parental neglect early in postnatal life and showed marked impairment in the ability of adult offspring to acquire contextual and spatial memory formation. It is generally thought that such cognitive deficits are mediated by a gradual deregulation of the stress axis following early life adversity. Intervention with anti-glucocorticoids directly targeted at the stress axis during a critical prepubertal developmental window completely normalized the behavior, possibly by targeting glutamatergic transmission in areas that are key for the behavior. Intervention strategies after the occurrence of maternal deprivation (MD) is highly relevant to the human situation, and may open new avenues for managing adverse consequences of early life stress.

## Introduction

Early life adversity is a well-recognized risk factor for the susceptibility of humans to mood and anxiety disorders (Kessler et al., 2010). The structural, physiologic and behavioral deficits developing after early life adversity have been studied in detail in various rodent models, including 24-h deprivation of rat pups of their mother (Maniam et al., 2014). Both in rats and mice exposed to early life adversity, contextual learning and memory in adulthood, depending on hippocampal function, was found to be impaired (Kosten et al., 2012), particularly in male animals (for review, see Loi et al., 2017). The potential underlying neuronal substrate, i.e., structural and functional plasticity in the adult hippocampus, was also altered in male animals with a history of early life adversity (Fenoglio et al., 2006). In humans, a disturbed ability to efficiently process contextual information may contribute to increased vulnerability to develop psychopathology when experiencing multiple life events (Anacker et al., 2014).

In addition to structural, physiologic and behavioral deficits reported after early life adversity, the functionality of the hypothalamo-pituitary-adrenal (HPA) axis, which is activated after stress, is known to be sensitive to the early life environment of an individual (Pryce et al., 2005). For instance, low licking-grooming behavior of the mother or unpredictability of maternal behavior due to impoverished housing conditions are known to raise levels of the stress hormones CRH and corticosterone (CORT), and alter expression of corticosteroid receptors in male rats, particularly glucocorticoid receptors (GRs; Weaver et al., 2006). In view of the well-established effects of stress hormones, such as corticosteroids, on cognitive function (Aisa et al., 2007; Lupien et al., 2009), it is conceivable that a dysfunctional HPA axis contributes to the development of behavioral deficits and thus may form a target for intervention.

To test this idea, we exposed rats to 24-h maternal deprivation (MD) on postnatal day (PND)3, examined their ability to form hippocampus-dependent contextual memories in young adulthood (approximately three months of age) and examined whether transient mifepristone (MIF) treatment, which among others blocks GRs, (van Haarst et al, 1996) at PND26-PND28 normalizes the expected behavioral deficits. This prepubertal period is important for priming the HPA axis and induces lasting changes in limbic function (Tsoory and Richter-Levin, 2006; Tzanoulinou et al., 2014). Moreover, MIF treatment during prepuberty was recently reported to prevent fear memory deficits of mice after early life stress (Arp et al., 2016). We also tested rats in a maze-based rodent model of the Iowa Gambling Task (rIGT; van den Bos R et al., 2006). This rodent task involves elements of hippocampus-striatal-based spatial learning (Yin and Knowlton, 2006) and prefrontal-striatal-based reward learning (for review, see de Visser et al., 2011a; van den Bos et al., 2014). To underpin the behavioral observations, we examined neuronal activity by c-Fos staining in brain structures likely contributing to the behavioral tasks, particularly hippocampal, prefrontal, and striatal areas (van den Bos et al., 2014). This guided subsequent electrophysiological investigation of spontaneous glutamatergic transmission in the dorsal hippocampus and dorsomedial striatum.

## Materials and Methods

### Animal breeding and housing

**Figure 1.**
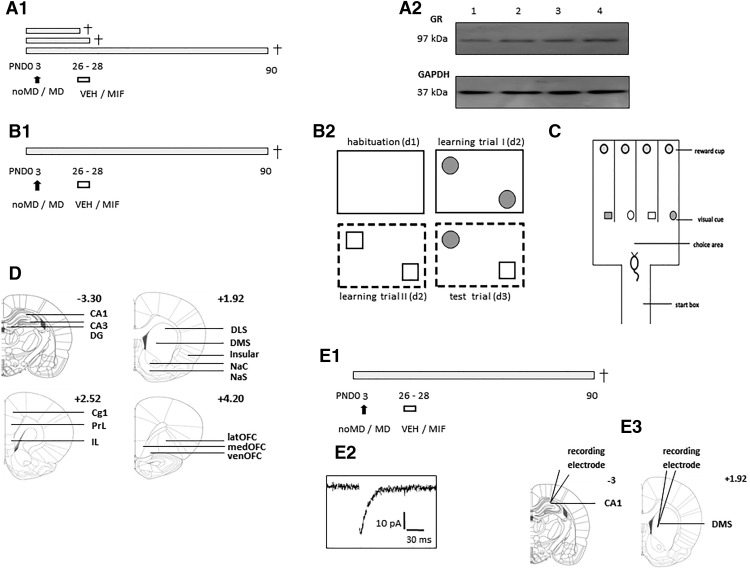
Schematic overview of the experimental design in this study. ***A***, Timeline for experimental series in which neuroendocrine data were collected (***A1***), either at PND26, PND29, or PND90. Animals were exposed to 24-h MD or control treatment at PND3. Part of the animals were treated twice daily with MIF or VEH administered through oral gavage directly into the stomach. At the indicated moment, we monitored body weight, adrenal weight (only at PND29), CORT levels, and collected hippocampal lobes for later Western blotting for MR and GR (typical examples shown in ***A2***). ***B***, Timeline for the object-in-(novel) context testing (***B1***). The details of the task are shown in ***B2***, see Materials and Methods for details. ***C***, Schematic representation of the rIGT maze model. ***D***, Anatomic localization of brain region used for analysis of c-Fos expression. Bregma coordinates are indicated above each coronal section. DG, dentate gyrus; CA1, CA1 region of the hippocampus; CA3, CA3 region of the hippocampus; DSL, dorsolateral striatum; DMS, dorsomedial striatum; NaC, nucleus accumbens core; NaS, nucleus accubmens shell; Cg1, cingulate cortex; PrL, prelimbic cortex; IL, infralimbic cortex; INS, insular cortex; medOFC, medial orbital frontal cortex (OFC); latOFC, lateral OFC; venOFC, ventral OFC. ***E***, Timeline for the electrophysiological recordings of CA1 pyramidal cells in hippocampal slices (***E1***). Typical example traces of mEPSCs are shown in ***E2***, at a low time resolution (left) and a high time resolution (right). ***E3***, Anatomic localization of brain region used for mEPSCs recording.

Before the start of the study, all animal procedures were approved by the animal ethics committee at Utrecht University, The Netherlands. All efforts were made to minimize suffering. Adult male and female Wistar rats were purchased from Charles River and habituated in pairs to the animal facilities for two weeks. For breeding, male rats were put together with female rats in a 1:2 ratio for a period of 10 d. Females were housed in pairs after mating until the last week of pregnancy when they were housed individually. Every morning before 9 A.M., cages were checked for births; on birth this day was denoted as PND0 (for experimental design, see Fig. 1). Dams with litters were left undisturbed until PND3.

On the morning of PND3, litters were randomly assigned to either the MD condition or the control (nonmaternally deprived, noMD) group. In the MD group, litters were separated from their mother after being culled; in the noMD group litters were merely culled. Litters, after being culled contained on average 9 ± 1 pups, of which four to five were females. For MD, the mother was placed in a separate cage; pups were returned to their home cage and placed on a heating pad (32°C) in a separate room. MD litters were kept in this room for 24 h before being placed back with the dam, as described elsewhere (Oomen et al., 2011). No animals died during the MD procedure.

On PND21, the litters were weaned and housed in same-sex groups of either two or three males or females. Here, we only focus on male rats. MD and noMD animals were then randomly assigned to treatment from PND26 through PND28 with MIF (5 mg/100 g bodyweight) or its vehicle (VEH) leading to four groups: noMD-VEH, MD-VEH, noMD-MIF, and MD-MIF. The age at which this intervention was applied was based on (1) the higher translational relevance of treating individuals after instead of during early life adversity; and (2) earlier studies showing that peripubertal activation of the stress system is highly effective in inducing long-lasting changes in limbic cells (Tzanoulinou et al., 2014) and priming organisms for their responsiveness to stress later in life (Horovitz et al., 2012). The duration of the intervention was based on earlier studies testing the potential of MIF to normalize effects of chronic stress in adulthood. A period of 1–4 d of treatment was found to be sufficient to completely reverse multiple effects of chronic stress (Mayer et al., 2006; Hu et al., 2012).

Per rat, 4 mg of MIF powder (Corcept Pharmaceuticals) was dissolved in 15 μl of 99% ethanol mixed with 1 ml of coffee cream (Campina) at body temperature, as described elsewhere (Hu et al., 2012). Twice daily, the drugs were administered by oral gavage (6–8 h in-between morning and late afternoon injections). To minimize litter effects, offspring from each MD or noMD litter were assigned to MIF and VEH groups.

All animals were kept under standard housing conditions (12/12 h light/dark cycle, lights on at 8 A.M., humidity 55 ± 15%, temperature 20–22°C) and received food and water ad libitum. They were weekly handled for 1 min, at which time point body weight was registered.

### Neuroendocrine parameters

In order to assess changes in neuroendocrine parameters a total of *n* = 80 male rats subjected to MD/noMD and receiving MIF or its VEH were included in this study. Figure 1*A1* provides an outline of the experiment. At the designated time points (PND26, PND29, or PND90), the animals were killed by decapitation. Decapitation always took place in the morning between 9:30 A.M. and 12 P.M. The bodyweight for each animal was determined immediately before decapitation. Trunk blood from all rats was collected to determine basal CORT levels, using a commercially available radioimmunoassay (MP Biomedicals). In case of two animals per cage, only one rat was randomly taken; in case of three rats, two rats were taken, since it has been shown that removal of rats from the home cage affects the CORT level of the remaining rat even at such short intervals (van Hasselt et al., 2012a). In those groups where this led to group sizes of less than five rats, we added basal CORT values in two to three randomly chosen rats from another cohort treated in the same manner. The brain was removed immediately after decapitation. The dorsal hippocampus, dorsal striatum and ventromedial prefrontal cortex (areas of interest in view of the behavioral tasks applied in this study) were dissected using a brain matrix, for later determination of GR expression by experimenters blinded to the condition.

### Western blotting

The dissected brain areas were homogenized in radioimmunoprecipitation assay lysis buffer (50 mM Tris HCl, 150 mM NaCl, 0.5% sodium deoxycholate, 0.1% SDS, 1% Triton X-100, and 2 mM EDTA). Per 5-mg tissue, 300 µl of lysis buffer was added. All samples were incubated in lysis buffer on ice for 30 min and mixed every 10 min using vortex. Protein samples were stored at −20°C until further processing.

Protein concentration was determined by bicinchoninic acid assay (Pierce BCA Protein Assay kit, Thermo Scientific). Proteins were denatured in LDS sample buffer (4 µl/sample; Novex) containing 10% β-mercaptoethanol and heated for 10 min at 70°C. Samples were separated in an 8% polyacrylamide gel at 25 mA/gel for ∼15 min until the samples reached the running gel, hereafter the current was speeded up to 35 mA/gel for ∼1 h until the samples passed the running gel. Migration of proteins was performed in a Tris/glycine/SDS running buffer. Subsequently the protein samples were transferred to a 0.45 µm nitrocellulose membrane in Tris/glycine/methanol (20%) transfer buffer at 100 V for 1 h. Afterward, the membrane was blocked in 5% dry powdered nonfat milk (Elk, Campina) dissolved in TBS for 1 h at room temperature. Following the blocking, membranes were probed with primary antibodies (anti-GR antibody rabbit GR M-20, Santa Cruz, 1:1000 dilution; anti-GAPDH antibody rabbit GAPDH, Cell Signaling, 1:3000 dilution) at 4°C overnight. Primary antibodies were diluted in 0.05% TBS-Tween (TBS-T). On the next day, membranes were washed with TBS-T (three times, 10 min each) and incubated with secondary antibodies (goat anti-rabbit IgG-HPR conjugate #170-6515, Bio-Rad and goat anti-mouse IgG-HPR conjugate #170-6516, Bio-Rad, both at 1:1000 dilution) for 1 h at room temperature. Secondary antibodies were diluted in 2% dry nonfat milk powder dissolved in TBS-T. After the second incubation, membranes were again washed in TBS-T (three times, 10 min each). For signal detection, membranes were incubated in Super Signal West Dura Extended Duration Substrate (Thermo Scientific) for 5 min at room temperature. Signal was detected using an enhanced chemiluminescence reader (Thermo Scientific). GAPDH was used as a loading control for total protein samples (Fig. 1*A2*).

Protein bands were expected at ∼37 kDa (GAPDH) and 97 kDa (GR). Signal density was analyzed with Image Studio Lite 5.0 software (LiCor) and subsequently corrected for background noise and control signal, and standardized to values in the noMD/VEH group at PND26 as reference group. Each Western blotting contained a complete time course of all experimental groups and ages, such that each sample point could be analyzed as fold inductions of basal values from the reference point (PND26 noMD/VEH). This allows comparison over time itself and within each group due to treatment and/or condition. All samples were processed and run at the same time allowing comparison after analysis. The values in the table depict mean ± SEM for fold inductions from all the pooled data sets.

### Behavioral paradigms

#### Object-in-context memory

In total, 31 male rats subjected to MD or noMD and receiving MIF or VEH were tested for contextual memory at approximately PND90 (Fig. 1*B1*), by experimenters blinded to the condition. Contextual memory was tested in an object-in-context task (Kanatsou et al., 2015; Fig. 1*B2*). The arenas were made of 2 identical solid plastic boxes (dimensions: 75 × 44 × 32 cm; w × l × h). On day 1, the habituation session, the rat was placed for 10 min in a box without objects. On day 2, the rat was placed for 10 min in a box (context A) that had no cues on the walls but contained two identical objects, i.e., two plastic pink cups, placed in opposite corners. Immediately thereafter, the rat was placed for 10 min into another box (context B) with black cues on the walls in the form of stripes and two (new) identical objects, i.e., two glass-staining transparent jars, placed in opposite corners. On day 3, the retention day, object-in-context recognition memory was tested by placing the rat for 5 min in context B. Context B on day 3 contained one object which also belonged to context B on day 2 (i.e., familiar object to context B), while the other object was earlier only encountered by the rat in context A. This object-in-novel-context-B is expected to raise the innate curiosity of the rat and to be visited more frequently and for longer duration during the 5-min retention test than the object already present in context B before. Based on pilot studies (data not shown), effects in this version of the object-in-novel context task were most clear when analyzing the entire 5-min retention period. The set-up (initial context and objects) was counterbalanced across rats. The objects were placed at a 15-cm distance from the corners and were cleaned between tests with unscented detergent diluted in water. Fresh bedding material was added on top of the old and mixed between each session.

All sessions were recorded using an infrared-sensitive camera. Videos were later analyzed manually using Observer XT 9 (Noldus) by experimenters blinded to the condition. Exploration of an object was defined similarly to other studies (Akkerman et al., 2012) as time spent attending to the object within a distance of 2 cm, focusing on the time spent exploring the object (duration). The discrimination ratio was determined by dividing the time spent with the object in the novel context by total exploration for the given variable. Animals had learned the task if the discrimination ratio was significantly different from 0.5 (=chance level). Sniffing was scored as object-exploration behavior if the rat displayed such behavior toward an object within a distance of <2 cm. Climbing on top of the objects was not considered as sniffing behavior.

#### rIGT

In total, 48 male rats (different from the ones tested for contextual memory) subjected to MD or noMD and receiving MIF or VEH entered the rodent version of the IGT (rIGT) approximately at PND90; experimenters were blinded to the condition. Group sizes (*n* = 12 per group) for the rIGT were calculated based on an earlier study (Koot et al., 2013), describing impaired reward-based performance on the final day of testing following acute CORT administration, with a moderate effect size (*d* = 0.6658). Animals were tested in two separate batches due to experimental constraints; each batch included rats randomly assigned to one of four experimental groups. *Post hoc* inspection revealed that task-performance was similar across batches; hence, data from the batches were pooled.

The protocol was based on earlier studies (Koot et al., 2013). First, each rat was subjected to the elevated plus maze (EPM) at week 10, to measure levels of anxiety. The EPM was made of gray plastic. The maze itself was elevated 60 cm from the ground and features two open arms (50 cm long × 10 cm wide) and two enclosed arms (50 cm long × 10 cm wide, walls 40 cm high) placed across from their respective counterparts. At the start, rats were placed in the middle, facing the open arms. Open arms were lit at 15 lux each, the middle part at 10 lux, and the two closed arms at 5 lux (Le Merrer et al., 2013; Van der Kooij et al., 2015; Fodor et al., 2016). Rats were allowed to freely explore the maze for 5 min. The behavior of the rats was recorded by a video camera and manually analyzed. We assessed the duration in the open and closed arms. Absolute data of the time spent in the arms was cumulated over arm categories (open or closed). From this a ratio was calculated, dividing the duration in the open (or closed) arm divided by the total.

One week later, rats were moved to another room and housed under a reversed day-night cycle (lights off at 8 A.M.); following an acclimation period of at least 10 d, they entered the rIGT. The rIGT apparatus consisted of a start box, choice area and four arms (Fig. 1*C*; for details, see de Visser et al., 2011a; van den Bos et al., 2014). Before testing, rats were habituated to the apparatus in a 10 min free exploration trial. Two days later, they were mildly food deprived (90–95% of free feeding body weight) and tested for a period of 10 d (two 5-d periods, 9 A.M. to 3 P.M., on weekdays; food freely available on weekend days).

Two arms were baited and two arms were empty; the latter were included to measure reward-unrelated exploration and potential spatial learning deficits (de Visser et al., 2011a; van den Bos et al., 2014). The two baited arms consisted of a long-term disadvantageous arm (“bad arm”) and a long-term advantageous arm (“good arm”). In the disadvantageous arm, rats received occasional big rewards (three sugar pellets in 1 out of 10 trials) among frequent punishments (three quinine-treated sugar pellets in nine out of 10 trials). In the advantageous arm, rats frequently received small rewards (one sugar pellet in eight out of 10 trials) and infrequent punishments (one quinine-treated sugar pellet in two out of 10 trials). The positions of the baited and empty arms, and the advantageous and disadvantageous arm, were counterbalanced across rats. To help rats differentiating arms, distinct visual cues (10 × 10 cm; cross or circle in black or white) were placed to the side of the wall at the entrance of each arm. The chosen arm was only closed when the rat had entered a choice arm with its full body, including the tail.

Each trial had a maximum duration of 6 min (intertrial interval: 30 s). Rats received a total of 120 trials across 10 d. Rewards consisted of 45-mg sugar pellets (F0042, Bio-Serv); punishments were quinine-treated sugar pellets, which were unpalatable but not inedible. Rats were habituated to the sugar pellets daily in the week before the first rIGT session in their home cage, followed by a single session of providing 6 sugar pellets in a novel empty Makrolon Type III cage; all rats consumed the pellets. During rIGT testing most rats consumed the quinine-treated sugar pellets once, and left them uneaten after tasting them briefly. Typically, rats that consistently eat quinine-treated sugar pellets are removed from statistical analyses; only one such rat was encountered in the current study and removed from further analyses (group noMD-VEH).

Experiments were done by someone blinded to which group the rats belonged. Following earlier studies (Koot et al., 2013) two different parameters were determined: the number of empty arm visits as a fraction of the total number of visits (per block of trials of interest) and the number of visits to the advantageous arms as fraction of the number of visits to the baited arms (per block of trials of interest). The former is a control for spatial learning/exploratory aspects, while the latter is related to reward learning (van den Bos et al., 2014). Performance in these two domains is not necessarily mutually linked; for instance, we have shown that manipulations may affect reward learning without affecting spatial learning per se (de Visser et al., 2011b; Koot et al., 2013, 2014). The data were expressed in blocks of 10 trials to study task progression. Scores in the last session were taken as a measure of final IGT performance of rats. The total number of sucrose pellets collected during the task (trial 1–120) was used as a measure of overall task performance and to reflect the final “budget.”

### c-Fos immunocytochemistry

Neuronal activity may dynamically change over the entire period of the task (12 d; van den Bos et al., 2014); here, we focused on the differences observed during the final day as described earlier (Koot et al., 2013). Measuring c-Fos immunoreactivity was primarily meant to assess which structures were of interest for further (electrophysiological) study, in line with earlier reports where we used the same approach to determine areas of interested for follow-up targeted microinjections (de Visser et al., 2011b; Koot et al., 2013).

Immunoreactivity was determined in fourteen areas important during the motivational and cognitive phases of the task (van den Bos et al., 2014; Koot et al., 2013). While c-Fos expression may not indicate functional activity, it is a powerful initial screen that may be followed up by experiments targeting structures of interest (for discussion of the rIGT, see van den Bos et al., 2014; Koot et al., 2013, 2014).

Two hours after the last session of the rIGT, rats were decapitated; their brains were quickly removed and frozen in liquid (−80*°C*) 2-methylbutane which was cooled by using dry ice. Brains were stored at −80*°C* until further processing. Coronal sections of 20 μm were cut on a cryostat, mounted on Menzel SuperFrost Plus slides (Menzel) and stored at −80°C. Immunohistochemical detection of c-Fos was performed according to the protocol previously published (Koot et al., 2013) using rabbit anti-c-Fos (Calbiochem). Anatomic localization of brain areas analyzed for c-Fos staining was based on the stereotaxic atlas of Paxinos and Watson (2005). For each region at least two overt landmarks were used, as shown in Figure 1*D*. Per experimental group an average of 9 sections per animals per area was analyzed. For quantitative analysis of c-Fos-positive cells, the program Leica QWIN (image processing and analysis software) was used. In view of lateralization effects of (chronic) stress, particularly in subregions of the PFC (Perez-Cruz et al., 2009), we analyzed the right and left hemispheres separately. The number of positive cells was then averaged for each animal and expressed per mm^2^. Out of 48 brains, six were excluded from the analysis due to low(er) quality of the sections. In all analysis steps, the experimenter was blinded to the condition.

### Electrophysiology

A separate batch of animals subjected to MD or noMD and receiving MIF or VEH was used for electrophysiological measurements. Animals were decapitated (at approximately PND90) within a few minutes after taking them from the homecage, which is short enough to not induce any discernable rise in plasma CORT concentration (Pasricha et al., 2011; for design, see Fig. 1*E1*). The brain was removed and kept in carbogenated (95% O_2_ and 5% CO_2_) artificial CSF (aCSF; 4°C) containing 120 mmol/l NaCl, 3.5 mmol/l KCl, 5.0 mmol/l MgSO_4_, 1.25 mmol/l NaH_2_PO_4_, 0.2 mmol/l CaCl_2_, 10 mmol/l D-glucose, and 25.0 mmol/l NaHCO_3_. Coronal slices containing either the dorsal hippocampus or striatum (350 µm) were cut on a Vibroslicer (Leica), stored at room temperature, and continuously gassed with carbogen until use.

One slice at a time was submerged in the recording chamber mounted on an upright microscope (Axioskop 2 FS plus; Zeiss) with differential interference contrast and a water immersion objective (40×) to identify the cells. The slices were continuously perfused (flow rate 1.5 ml/min, temperature 30°C, pH 7.4) with aCSF to which was added TTX (0.5 µM; Latoxan) to block sodium channels, and bicuculline (50 µM; Enzo) to block GABA_a_ receptors.

Patch pipettes (borosilicate glass pipettes, inner diameter 0.86 mm, outer diameter 1.5 mm; Hilgenberg) were pulled on a Sutter micropipette puller and had a tip resistance of 3–6 MΩ when filled with the pipette (intracellular) solution, containing 120 mM Cs methane sulfonate, 17.5 mM CsCl, 10 mM HEPES, 2 mM MgATP, 0.1 mM NaGTP, and 5 mM BAPTA; pH was 7.4, adjusted with CsOH. BAPTA was obtained from Invitrogen, all other chemicals were obtained from Sigma-Aldrich Chemie B.V. An Axopatch 200B amplifier (Axon Instruments) was used for whole cell recordings, operating in the voltage-clamp mode. The patch-clamp amplifier was interfaced to a computer via a Digidata (type 1200; Molecular Devices) analog-to-digital converter.

Routinely, we cleaned the surface of the slice. Only cells from the dorsal hippocampus were selected at the following anterior-posterior coordinates in mm from bregma: −1.94 to −2.18. After establishing a gigaseal, the membrane patch was ruptured and the cell was held at a holding potential of −70 mV. The liquid junction potential caused a shift of <8 mV, which was not compensated. Recordings with an uncompensated series resistance of <2.5 times the pipette resistance were accepted for analysis. Series resistances were typically between 6 and 15 MΩ, and if the series resistance changed by >10% with time or on application of the drug, the recording was not incorporated in the analysis. In view of the small current amplitudes, the recordings were not corrected for series resistance.

Data acquisition and storage was done with PClamp (version 9.2). The currents were recorded at a holding potential of −70 mV with the sampling rate set at 10 kHz and filters at 5 kHz. Miniature EPSCs (mEPSCs) were accepted if the rise time was faster than the decay time (Pasricha et al., 2011). In all cells measured, the following mEPSC characteristics were determined off-line with ClampFit 9.2: inter-mEPSC interval, the frequency, rise time, peak amplitude, and τ of decay between 5 and 10 min after establishing the whole cell recording configuration.

Data acquisition and analysis were done by experimenters blinded to the condition; the code was only revealed after analysis was completed.

### Statistical analysis

Neuroendocrine data at PND26 (before MIF treatment) were tested for statistical significance with an unpaired Student’s *t* test. In the rIGT and object-in-context test, data were first analyzed using one-sample *t* tests (vs 0.5: indicative of no learning). In the rIGT, we applied paired *t* tests for testing first trial block versus last trial block, as indicated in Results.

All other data were analyzed using two-way ANOVA, with rearing condition (MD vs noMD) and treatment (MIF vs VEH) as factors. In the rIGT, trial block was added as repeated measure leading to a three-way ANOVA. In case of baited arm scores: when rats only visited the empty arms in a block of 10 trials, data were missing for this parameter, leaving fewer subjects for statistical analysis.

Significant interaction (but not main) effects were followed up by *post hoc* LSD testing. *Post hoc* comparisons of interest were: MD-VEH versus noMD-VEH (to assess the effects of MD on learning), MD-MIF versus MD-VEH (to assess whether MIF restores the negative effects of MD if present) and noMD-MIF versus noMD-VEH (to assess whether MIF has effects by itself on learning). The cumulative distribution of mEPSC frequencies was compared with a Kolmogorov-Smirnov test (KS test).

All statistical analyses were conducted using SPSS version 20.0 (IBM). In all cases, *p* < 0.05 was considered to reflect significant differences, while *p* < 0.10 (two-tailed) was considered a trend.

## Results

### Neuroendocrine parameters

At PND26, i.e., before treatment, body weight was significantly lower in the MD compared to noMD group (Table I). Plasma CORT levels were significantly elevated in rats earlier exposed to MD, while GR protein levels in the ventromedial PFC but not the dorsal hippocampus or dorsal striatum were significantly increased.

**Table 1.** Body weight, basal CORT concentration, and GR expression (fold induction, standardized to the noMD group at PND26) in the dorsal hippocampus, ventromedial prefrontal cortex (vmPFC), and dorsal striatum of male rats at PND26, PND29, and PND90

	noMD		MD		Condition		
PND26							
Body weight (BW in g)	59 ± 1		52 ± 1		*t*_(78)_ = 6; *p* < 0.0001		
Basal CORT (ng/ml)	57 ± 20		178 ± 45		*t*_(22)_ = 5.7; *p* = 0.025		
GR hippocampus	1.0 ± 0.3		1.0 ± 0.2		*t*_(15)_ = 0.23; *p* = 0.140		
GR vmPFC	1.0 ± 0.2		2.4 ± 0.6		*t*_(13)_ = −2.2; *p* = 0.049		
GR striatum	1.0 ± 0.2		1.8 ± 0.6		*t*_(8)_ = 0.2; *p* = 0.749		
PND29	noMD VEH	noMD MIF	MD VEH	MD MIF	Condition × treatment	Condition (noMD vs MD)	Treatment (VEH vs MIF)
Body weight (BW in g)	75 ± 2	72 ± 1	65 ± 1	64 ± 2	*F*_(1,28)_ = 0.4; *p* = 0.532	*F*_(1,28)_ = 47; *p* < 0.0001	*F*_(1,28)_ = 1.9; *p* = 0.180
Basal CORT (ng/ml)	228 ± 60	122 ± 45	68 ± 34	115 ± 65	*F*_(1,20)_ = 2; *p* = 0.172	*F*_(1,20)_ = 2.4;*p* = 0.140	*F*_(1,20)_ = 0.3; *p* = 0.593
GR hippocampus	1.2 ± 0.2	0.8 ± 0.1	1.1 ± 0.3	1.2 ± 0.2	*F*_(1,27)_ = 1.5; *p* = 0.226	*F*_(1,27)_ = 0.35 *p* = 0.560	*F*_(1,27)_ = 0.41; *p* = 0.525
GR vmPFC	1.2 ± 0.3	1.9± 0.6	1.4 ± 0.4	1.1 ± 0.3	*F*_(1,24)_ = 1; *p* = 0.322	*F*_(1,24)_ = 0.4; *p* = 0.524	*F*_(1,24)_ = 3.7; *p* = 0.545
GR striatum	1.6 ± 0.7	2.2 ± 0.8	1.3 ± 0.3	1.4 ± 0.5	*F*_(1,20)_ = 0.2; *p* = 0.692	*F*_(1,20)_ = 0.8; *p* = 0.370	*F*_(1,20)_ = 0.3; *p* = 0.590
PND90	noMD VEH	noMD MIF	MD VEH	MD MIF	Condition × treatment	Condition (noMD vs MD)	Treatment (VEH vs MIF)
Body weight (BW in g)	329 ± 6	334 ± 6	333 ± 5	331 ± 8	*F*_(1,29)_ = 1.3; *p* = 0.262	*F*_(1,29)_ = 0.7; *p* = 0.415	*F*_(1,29)_ = 0.8; *p* = 0.368
Basal CORT (ng/ml)	191 ± 60	206 ± 56	104 ± 21	118 ± 37	*F*_(1,24)_ = 0.0; *p* = 0.991	*F*_(1,24)_ = 3.6; *p* = 0.071	*F*_(1,24)_ = 0.10; *p* = 0.753
GR hippocampus	1.4± 0.4	0.9 ± 0.3	1.4 ± 0.4	0.8 ± 0.2	*F*_(1,26)_ = 0.3; *p* = 0.871	*F*_(1,26)_ = 0.4; *p* = 0.834	*F*_(1,26)_ = 3.2; *p* = 0.9
GR vmPFC	2.2 ± 0.9	1.2 ± 0.3	1.2 ± 0.3	1.3 ± 0.5	*F*_(1,24)_ = 0.9; *p* = 0.350	*F*_(1,24)_ = 0.6; *p* = 0.430	*F*_(1,24)_ = 0.8; *p* = 0.375
GR striatum	1.1 ± 0.8	1.8 ± 1	1.3 ± 0.5	0.6 ± 0.2	*F*_(1,19)_ = 1.1; *p* = 0.300	*F*_(1,19)_ = 0.5; *p* = 0.505	*F*_(1,19)_ = 0.0; *p* = 0.994

All animals had been subjected to 24-h MD at PND3 or control treatment. Half of the animals were treated with MIF through oral gavage twice daily on PND26–PND28; the other half received VEH. Data represent mean ± SEM; all groups *n* = 6–8 animals, except for body weight at PND26, which was determined for all animals.

At PND29, i.e., 1 day following pharmacological treatments, body weight was significantly decreased due to MD, independent of MIF or VEH treatment (Table I). Basal CORT levels were rather variable and we no longer observed an effect of condition, nor of treatment (Table 1). GR protein expression in the tested brain areas were not significantly different between the groups (Table 1).

At PND90, i.e., at the time of behavioral testing, c-Fos staining and electrophysiology (in different batches of animals), no differences were found between groups for body weight, basal CORT or GR protein expression (Table 1).

Overall these data indicate that (1) some parameters were affected by MD just before MIF treatment, and (2) no effects of condition and/or treatment were found anymore at the time of behavioral or electrophysiological experiments.

### Object-in-context memory

The score of one animal from the noMD-VEH group was considered an outlier (>2 SD from the group mean); this subject was excluded from the final analysis.

**Figure 2.**
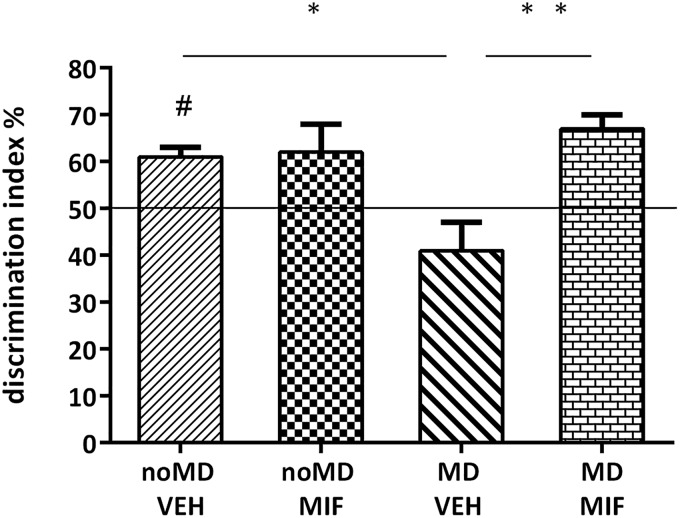
Object-in-context memory formation. Discrimination index in the retention test on day 3 (*n* = 7–8 animals per group). The discrimination index is based on the duration of the object in the novel context during the entire 5-min period. One-sample *t* test revealed that noMD-VEH (control) group discriminated between the object in the matching and the object in the nonmatching context as scores were significantly above chance level (50%; #*p* < 0.001). Two-way ANOVA revealed a significant condition × treatment interaction effect (see main text). *Post hoc* analysis revealed significant differences between the MD-VEH and MD-MIF as well as noMD-VEH groups. All bars represent the mean + SEM. #*p* < 0.001 (paired *t* test against chance level); *p < 0.05; **p < 0.01 (*post hoc* comparisons after ANOVA).

First, we checked whether the noMD-VEH (control) group learned the task. Rats from this group discriminated between the object in the matching and the object in the nonmatching context, as scores were significantly above chance level (50%; *p* < 0.001; one-sample *t* test; Fig. 2). A significant condition × treatment effect was found for duration (*F*_(1,27)_ = 4.6, *p* = 0.041) with which rats visited the object in the nonmatching compared to matching context. *Post hoc* comparison for duration revealed that MD-VEH rats performed significantly worse than noMD-VEH (*p* = 0.02) or MD-MIF rats (*p* = 0.004). NoMD-MIF rats did not differ significantly from noMD-VEH animals (Fig. 2).

### EPM

All animals tested in the rIGT were first examined in the EPM [percentage of time spent in open arm in noMD-VEH: 14.1 ± 3.5 (mean ± SEM); noMD-MIF: 17.8 ± 4.2; MD-VEH: 19.3 ± 4.8; MD-MIF: 19.2 ± 3.5]. We observed no significant condition × intervention interaction (*F*_(1,39)_ = 0.198, *p* = 0.659), nor main effects of condition (*p* = 0.439) or intervention (*p* = 0.673). This suggests that neither MD nor intervention had an effect on anxiety-like behavior as measured by EPM performance.

### rIGT

#### Empty arms

**Figure 3.**
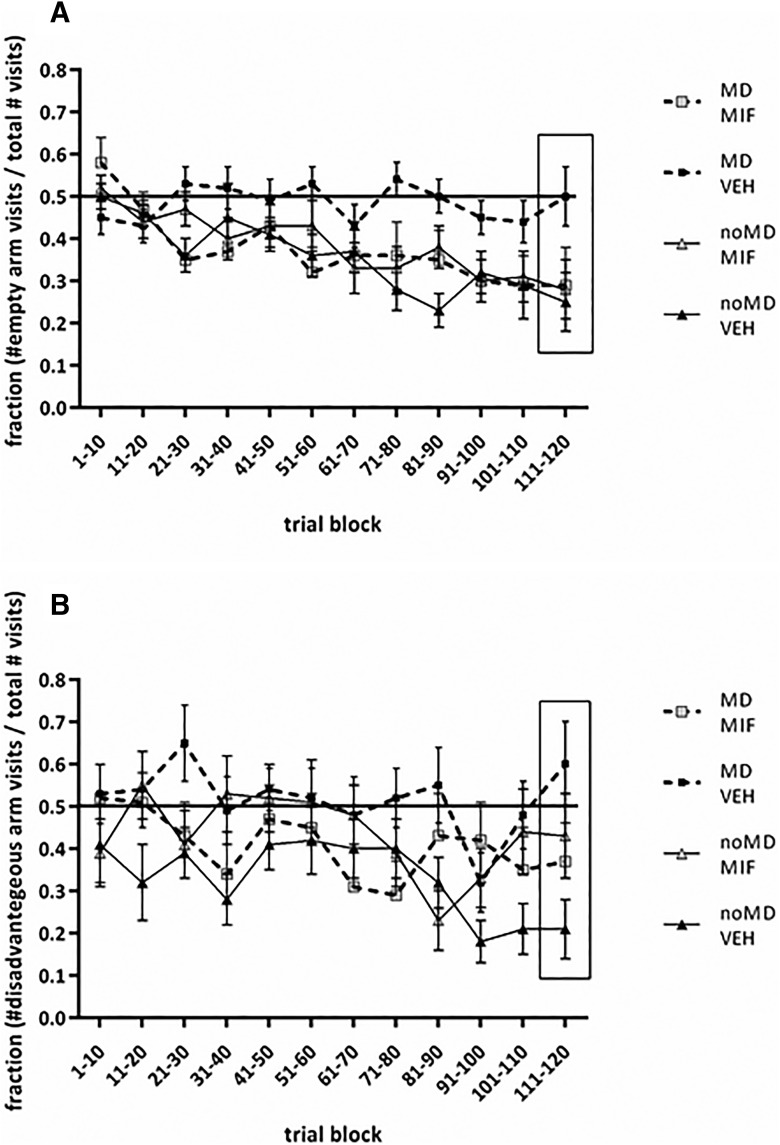
rIGT performance in male rats (PND90) exposed to MD or control treatment (noMD), receiving MIF or VEH between PND26 and PND28. All data were tested with a three-way ANOVA. For the parameters shown in ***A***, ***B***, we observed significant interaction effects of treatment and condition (see main text for statistical details). ***A***, Mean (±SEM) fraction of empty arms choices across 12 trial blocks (empty arm visits as fraction of the total number visits per trial block; *n* = 10). The inset in the figure shows the scores of the last block of trials: 111–120. ***B***, Mean (±SEM) fraction of disadvantageous arm choices across 12 trial blocks (disadvantageous arm visits as fraction of total number of visits to baited arms per trial block). The inset in the figure shows the scores of the last block of trials: 111–120.

One week after the EPM, the animals entered the rIGT. First, we assessed whether rats in the noMD-VEH (control) group learned to discriminate the empty arms from the baited arms. The scores were consistently and significantly lower than chance level (=0.5) from trial block 51–60 onwards (one-sample *t* test, *p* = 0.04–0.001; values in trial block 21–30 were also lower than chance: *p* = 0.006). In addition, scores in trial block 111–120 were significantly lower than in block 1–10 (*p* = 0.006). Both analyses indicate that rats learned to discriminate the empty from the baited arms (Fig. 3*A*). The data in Figure 3*A* show furthermore that, except for rats in the MD-VEH group, rats in the other groups learned to discriminate the empty arms from the baited arms, which was confirmed by statistical analysis. The performance of MD-VEH rats did not deviate from chance level (0.5; all *p* > 0.17), while those of MD-MIF (consistently and significantly lower from trial block 91–100 onwards: *p* = 0.04–0.001; trial block 21–30, *p* = 0.007; trial block 51–60, *p* = 0.01) and noMD-MIF (consistently and significantly lower from trial block 61–70 onwards: *p* = 0.04–0.001) did. For MD-VEH values of trial block 111–120 and 1–10 did not differ (*p* = 0.66), while they were lower for MD-MIF (*p* = 0.01) and noMD-MIF rats (*p* = 0.01). This difference between groups was further confirmed by a three-way ANOVA [factors: condition, treatment, and trial block (repeated)]: a significant overall trial block effect (*F*_(11,473)_ = 5.118, *p* < 0.001), a significant three way interaction (*F*_(11,473)_ = 1.912, *p* = 0.04), and a significant overall two-way condition × treatment interaction (*F*_(1,43)_ = 5.548, *p* = 0.02). Planned comparisons showed (1) that rats in the MD-VEH group did not learn to discriminate the empty arms from the baited arms while those of the noMD-VEH group did (condition × trial block: *F*_(11,231)_ = 2.419, *p* = 0.007; condition *F*_(1,21)_ = 23.749, *p* = 0.001), (2) that rats in the MD-MIF learned to discriminate the empty arms from the baited arms while MD-VEH rats did not (MD-MIF vs MD-VEH: treatment × trial block *F*_(11,242)_ = 2.069, *p* = 0.02; treatment *F*_(1,22)_ = 6.310, *p* = 0.02), and (3) that no significant differences were found between noMD-VEH and noMD-MIF (treatment * trial block *F*_(11,242)_ = 0.812, *p* = 0.63; treatment *F*_(1,21)_ = 0.482, *p* = 0.50). Overall these data indicate that MD-VEH rats did not learn to discriminate empty from baited arms, while all other rats did, and that MIF restored the deficit in the MD rats. As the c-Fos data are related to the last day of testing (10 trials), Figure 3*A*, box, shows the data from trial block 111–120 only. Statistical analysis showed a near significant interaction term (*F*_(1,43)_ = 3.867, *p* < 0.056), probably explained by the higher value of MD-VEH rats compared to all other groups in line with the findings above.

#### Baited arms

First, we checked whether the noMD-VEH (control) group learned to discriminate the advantageous arm from the disadvantageous arm in the baited arms. Scores were consistently and significantly lower than chance level (=0.5) from trial block 81–90 onwards (one-sample *t* test, *p* values = 0.01–0.0010; scores in trial block 31–40 were also lower: *p* = 0.004). However, scores of block 111–120 were not significantly lower than of block 1–10 (*p* = 0.18). Despite the latter (see Discussion), the overall results suggest that with regard to the baited arms rats learned to discriminate the advantageous from the disadvantageous (Fig. 3*B*). The data in Figure 3*B* suggest that scores of rats in the MD-VEH group did not deviate from chance level, while scores of the MIF-treated rats were variable suggesting an effect of MIF. Subsequent statistical analysis confirmed this impression. Apart from trial block 91–100 (*p* = 0.03) scores in the MD-VEH group were not different from chance level, with no difference between trial block 111–120 and 1–10 (*p* = 0.46). Apart from trial block 81–90 (*p* = 0.002) and 91–100 (*p* = 0.04) scores in the noMD-MIF group were not different from chance level with no difference between trial block 111–120 and 1–10 (*p* = 0.78). Apart from trial block 61–70 (*p* = 0.04) and 71–80 (*p* = 0.02) scores in the noMD-MIF group were not different from chance level, with no difference between trial block 111–120 and 1–10 (*p* = 0.10). A three-way ANOVA showed, next to a significant overall trial block effect (*F*_(11,451)_ = 2.317, *p* = 0.009), a significant overall two-way condition * treatment interaction: *F*_(1,41)_ = 4.456, *p* = 0.04. Subsequent planned comparisons revealed (1) that scores of MD-VEH rats were higher than of noMD-VEH rats (treatment: *F*_(1,19)_ = 10.203, *p* = 0.005), (2) that scores of MD-MIF rats did not differ from scores of MD-VEH rats (all *p* > 0.22), and (3) that scores of noMD-MIF rats tended to be higher than of noMD-VEH rats (treatment: *F*_(1,20)_ = 3.454, *p* = 0.08). Overall, these data indicate that MD-VEH rats did not learn to discriminate the advantageous arm from the disadvantageous arm in the baited arms, while noMD-VEH rats did; in addition, MIF did not restore the deficit in the MD rats and may have had an effect per se. As the c-Fos data are related to the last day of testing (10 trials), Figure 3*B*, box, shows the data from trial block 111–120. Statistical analysis showed a significant condition × treatment interaction term (*F*_(1,43)_ = 5.722, *p* < 0.02): scores of MD-VEH rats were found to be higher than of noMD-VEH rats (*p* = 0.006), while scores of MIF-treated rats were in-between the scores of these groups in line with the findings above.

As a consequence of impaired learning (empty arms vs baited arms visits, and advantageous vs disadvantageous visits) rats in the MD-VEH group earned overall significantly fewer sugar pellets (397 ± 2.17) than rats in the noMD-VEH group (548 ± 2.95; *p* = 0.0001) Table 2; since all rats did eat pellets, it seems unlikely that the reduction in number of pellets can be explained by anhedonia. The reduction was restored by treatment with MIF in the MD group (544 ± 5.08; *p* = 0.0001).

**Table 2.** Performance in a rodent version of the rIGT

	noMD VEH	noMD MIF	MD VEH	MD MIF	Condition × treatment	Condition (noMD vs MD)	Treatment (VEH vs MIF)
#Sugar pellets gained	548 ± 2.95	521 ± 3.77	397 ± 2.17	544 ± 5.08	*F*_(1,44)_ = 3.9; *p* = 0.054	*F*_(1,44)_ = 2.1; *p* = 0.152	*F*_(1,44)_ = 1.9; *p* = 0.178
#Switches 2nd half	5.61 ± 0.23	6.06 ± 0.24	6.25 ± 0.28	4.72 ± 0.27	*F*_(1,44)_ = 4.5; *p* = 0.040	*F*_(1,44)_ = 0.6; *p* = 0.459	*F*_(1,44)_ = 1.4; *p* = 0.250
Win-stay (total)	0.15 ± 0.20	0.13 ± 0.19	0.05± 0.07	0.18 ± 0.24	*F*_(1,44)_ = 4.2; *p* = 0.046	*F*_(1,44)_ = 0.4; *p* = 0.508	*F*_(1,44)_ = 1.8; *p* = 0.192

All animals had been subjected to 24-h MD at PND3 or control treatment. Half of the animals were treated with MIF through oral gavage twice daily on PND26–PND28; the other half received VEH. Data represent mean ± SEM; all groups *n* = 11-12 animals. Visits to the arm are expressed as a fraction of the total number of trials.

### c-Fos labeling

Table 3 shows the data of c-Fos expression in different brain areas; sections of six brains (of *n* = 48 total) were excluded from analyses due to poor quality of sections. First, we observed significant condition × treatment interactions for the right dorsal CA1 and CA3 hippocampal regions, right insular cortex, right dorsomedial striatum and right nucleus accumbens shell. In particular, in the right CA1 and CA3 region of the hippocampus c-Fos expression in the MD-VEH group was lower than in the noMD-VEH group and the MD-MIF group. By contrast, in the right dorsomedial striatum, nucleus accumbens shell and insular cortex c-Fos expression was on average higher in the MD-VEH group compared to the noMD-VEH and MD-MIF groups. Of these, significant *post hoc* effects were demonstrated in the right dorsomedial striatum (MD-VEH > MD-MIF, *p* = 0.02, all other comparisons ns); and in the right CA3 region (MD-VEH < noMD-VEH, *p* = 0.02). Second, we observed significant main effects of rearing condition (MD lower than noMD) for the right dentate gyrus of the hippocampus, the left cingular cortex and left prelimbic cortex, suggesting that MD per se but not MIF treatment affected these areas.

**Table 3.** Immunoreactivity for c-Fos (number of labeled cells/mm^2^), 2 h after the final session in the rIGT

	noMD VEH	noMD MIF	MD VEH	MD MIF	Condition × treatment	Condition (noMD vs MD)	Treatment (VEH vs MIF)
l-DG	100 ± 49.9	97 ± 20.5	141.2 ± 54	55.8 ± 16.7	*F*_(1,21)_ = 0.8; *p* = 0.387	*F*_(1,21)_ = 0.0; *p* = 1	*F*_(1,21)_ = 0.9; *p* = 0.354
r-DG	100 ± 55.3	64.3 ± 17	32.6 ± 5.3	22.7 ± 5.6	*F*_(1,24)_ = 0.3; *p* = 0.594	*F*_(1,24)_ = 5.2; *p* = 0.032	*F*_(1,24)_ = 0.9; *p* = 0.348
l-CA1	100 ± 43.6	110.5 ± 32.9	93 ± 28	85 ± 20.9	*F*_(1,33)_ = 0.0; *p* = 0.779	*F*_(1,33)_ = 0.2; *p* = 0.662	*F*_(1,33)_ = 0.0; *p* = 0.969
r-CA1	100 ± 27	44.9 ± 16	30.5 ± 6.6	77.5 ± 17.4	*F*_(1,30)_ = 7.7; *p* = 0.009	*F*_(1,30)_ = 1.0; *p* = 0.324	*F*_(1,30)_ = 0.0; *p* = 0.828
l-CA3	100 ± 22.4	111.9 ± 32.5	93.1 ± 18.9	285.4 ± 18.7	*F*_(1,27)_ = 1.1; *p* = 0.239	*F*_(1,27)_ = 1.2; *p* = 0.276	*F*_(1,27)_ = 1.8; *p* = 0.184
r-CA3	100 ± 29.3	42.1 ± 9.7	28.9 ± 6.2	53.2 ± 12.8	*F*_(1,30)_ = 6.1; *p* = 0.018	*F*_(1,30)_ = 3.3; *p* = 0.079	*F*_(1,30)_ = 1.0; *p* = 0.318
l-DLS	100 ± 35.6	136 ± 51.2	131 ± 36.4	70 ± 13.4	*F*_(1,33)_ = 1.6; *p* = 0.215	*F*_(1,33)_ = 0.2; *p* = 0.654	*F*_(1,33)_ = 0.1; *p* = 0.737
r-DLS	100 ± 40.3	106.4 ± 16.3	117.3 ± 36.1	52.4 ± 9.3	*F*_(1,34)_ = 1.7; *p* = 0.193	*F*_(1,34)_ = 0.5; *p* = 0.500	*F*_(1,34)_ = 1.2; *p* = 0.284
l-DMS	100 ± 21.1	61.6 ± 16.6	69 ± 11	47 ± 9.7	*F*_(1,29)_ = 0.3; *p* = 0.596	*F*_(1,29)_ = 2.3; *p* = 0.142	*F*_(1,29)_ = 4.0; *p* = 0.052
r-DMS	100 ± 29.9	111.4 ± 26	236.1 ± 79.3	45.2 ± 7.2	*F*_(1,33)_ = 4.6; *p* = 0.040	*F*_(1,33)_ = 0.5; *p* = 0.466	*F*_(1,33)_ = 3.6; *p* = 0.067
l-Insular	100 ± 17.4	75.3 ± 17.6	50.9 ± 16	54 ± 15.2	*F*_(1,32)_ = 0.6; *p* = 0.434	*F*_(1,32)_ = 4.0; *p* = 0.054	*F*_(1,32)_ = 0.4; *p* = 0.542
r-Insular	100 ± 17	125.4 ± 23.5	128.6 ± 30.1	51.6 ± 9.3	*F*_(1,35)_ = 4.9; *p* = 0.033	*F*_(1,35)_ = 0.9; *p* = 0.335	*F*_(1,35)_ = 1.2; *p* = 0.272
l-NaC	100 ± 24.3	89 ± 20.9	145.2 ± 28	86.6 ± 18.2	*F*_(1,32)_ = 0.9; *p* = 0.346	*F*_(1,32)_ = 0.7; *p* = 0.400	*F*_(1,32)_ = 1.9; *p* = 0.172
r-NaC	100 ± 34.1	85.9 ± 23	87.1 ± 23.9	65.4 ± 14.4	*F*_(1,31)_ = 0.3; *p* = 0.872	*F*_(1,31)_ = 0.5; *p* = 0.484	*F*_(1,31)_ = 0.6; *p* = 0.453
l-NaS	100 ± 33.5	57.9 ± 16.1	74.7 ± 19.2	50.8 ± 9.5	*F*_(1,33)_ = 0.2; *p* = 0.640	*F*_(1,33)_ = 0.7; *p* = 0.407	*F*_(1,33)_ = 2.9; *p* = 0.097
r-NaS	100 ± 28.4	147 ± 27.1	195.1 ± 53.7	72.5 ± 17	*F*_(1,32)_ = 5.2; *p* = 0.029	*F*_(1,32)_ = 0.0; *p* = 0.793	*F*_(1,32)_ = 1.0; *p* = 0.323
l-Cg1	100 ± 25.1	165.9 ± 41	52.2 ± 7.6	40.9 ± 9.7	*F*_(1,27)_ = 2.4; *p* = 0.133	*F*_(1,27)_ = 12; *p* = 0.002	*F*_(1,27)_ = 1.2; *p* = 0.282
r-Cg1	100 ± 25	132.8 ± 22.9	94.3 ± 20.2	88 ± 15.2	*F*_(1,30)_ = 0.8; *p* = 0.352	*F*_(1,30)_ = 1.5; *p* = 0.232	*F*_(1,30)_ = 0.4; *p* = 0.528
l-Prl	100 ± 29.2	92.7 ± 25.6	44.6 ± 10.2	59.8 ± 8.5	*F*_(1,27)_ = 0.3; *p* = 0.558	*F*_(1,27)_ = 5.3; *p* = 0.029	*F*_(1,27)_ = 0.4; *p* = 0.837
r-Prl	100 ± 22.6	196.3 ± 38.5	119.1 ± 35.6	114.1 ± 12.8	*F*_(1,30)_ = 3.2; *p* = 0.081	*F*_(1,30)_ = 1.2; *p* = 0.269	*F*_(1,30)_ = 2.6; *p* = 0.114
l-IL	100 ± 17.6	80.2 ± 27.3	44.9 ± 10.9	78.3 ± 9.8	*F*_(1,24)_ = 2.5; *p* = 0.125	*F*_(1,24)_ = 2.9; *p* = 0.102	*F*_(1,24)_ = 0.2; *p* = 0.688
r-IL	100 ± 29.8	114.4 ± 21.7	112.7 ± 39.2	107.6 ± 20.6	*F*_(1,31)_ = 0.1; *p* = 0.737	*F*_(1,31)_ = 00; *p* = 0.919	*F*_(1,31)_ = 0.0; *p* = 0.873
l-latOFC	100 ± 17.2	88.9 ± 19.1	81 ± 10.4	82.2 ± 18	*F*_(1,31)_ = 0.1; *p* = 0.735	*F*_(1,31)_ = 0.5; *p* = 0.484	*F*_(1,31)_ = 0.0; *p* = 0.788
r-latOFC	100 ± 7	88 ± 12.4	79.8 ± 12.2	92.1 ± 16.7	*F*_(1,29)_ = 0.8; *p* = 0.383	*F*_(1,29)_ = 0.3; *p* = 0.562	*F*_(1,29)_ = 0.0; *p* = 0.991
l-medOFC	100 ± 24.6	87.4 ± 13.6	117.1 ± 17.2	129.3 ± 29.3	*F*_(1,30)_ = 0.3; *p* = 0.580	*F*_(1,30)_ = 1.8; *p* = 0.192	*F*_(1,30)_ = 0.0; *p* = 0.992
r-medOFC	100 ± 18.5	132.6 ± 25.1	77.5 ± 15.1	118.8 ± 30.6	*F*_(1,31)_ = 0.0; *p* = 0.853	*F*_(1,31)_ = 0.6; *p* = 0.442	*F*_(1,31)_ = 2.5; *p* = 0,124
l-venOFC	100 ± 13	82.4 ± 15.4	85.1 ± 9.4	103.7 ± 25.1	*F*_(1,31)_ = 1.0; *p* = 0.322	*F*_(1,31)_ = 0.0; *p* = 0.860	*F*_(1,31)_ = 0.0; *p* = 0,977
r-venOFC	100 ± 8.5	95.6 ± 10.6	80.8 ± 7.6	96 ± 15.7	*F*_(1,31)_ = 0.6; *p* = 0.432	*F*_(1,31)_ = 0.5; *p* = 0.449	*F*_(1,31)_ = 0.2; *p* = 0,665

After completion of the analysis, all data were normalized to the noMD-VEH group. Experimental animals had been subjected to 24-h MD at PND3 or control treatment. Half of the animals were treated with MIF through oral gavage twice daily on PND26–PND28; the other half received VEH. Data represent mean ± SEM; all groups *n* = 9-12 animals. l, left; r, right; DG, dentate gyrus; CA1, CA1 region of the hippocampus; CA3, CA3 region of the hippocampus; DSL, dorsolateral striatum; DMS, dorsomedial striatum; NaC, nucleus accumbens core; NaS, nucleus accumbens shell; Cg1, cingulate cortex; PrL, prelimbic cortex; IL, infralimbic cortex; INS, insular cortex; medOFC, medial orbital frontal cortex (OFC); latOFC, lateral OFC; venOFC, ventral OFC.

### Glutamatergic transmission in hippocampus and dorsomedial striatum

The fact that CA areas of the hippocampus and dorsomedial striatal areas are connected and involved in flexible spatial learning (Devan et al., 2011; Fouquet et al., 2013; Delcasso et al., 2014), combined with the observed effects on spatial/contextual learning of MD, their reversal by MIF treatment and the c-Fos immunoreactivity data, prompted us to study changes in excitability in these areas. In particular, we measured mEPSCs, as index of spontaneous glutamatergic transmission, in pyramidal neurons of the dorsal CA1 hippocampal area and medium spiny neurons in the dorsomedial striatum, identified through the recording microscope.

**Figure 4.**
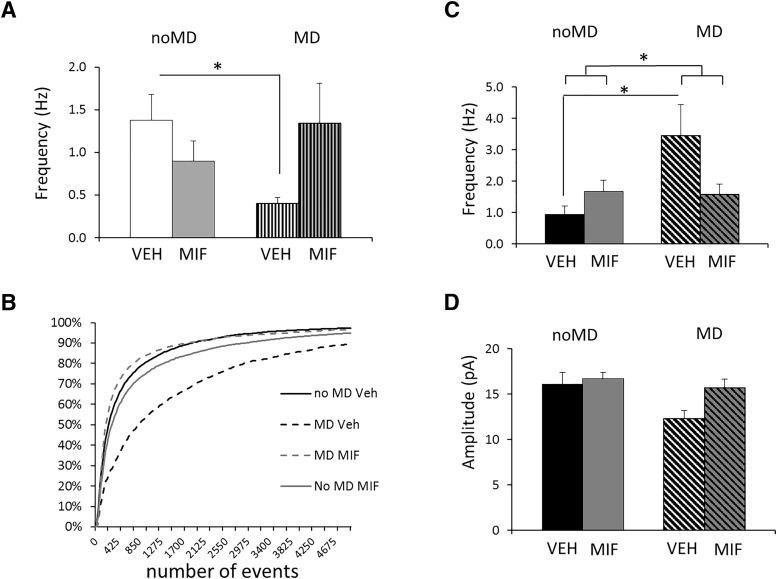
Changes in hippocampal and striatal mEPSCs frequency after MD are normalized by MIF treatment. ***A***, The frequency of mEPSCs recorded in adult CA1 pyramidal neurons *in vitro* was found to be significantly reduced after 24-h MD experienced at PND3. This was normalized when animals were subjected to MIF treatment between PND26 and PND28, as opposed to VEH treatment. The data show the averaged mEPSC frequency ± SEM in a period of 5–10 min after establishing the whole cell recording configuration. Amplitude was not affected by either factor. Group sizes: *n* = 16 cells in noMD/VEH; *n* = 21 in noMD/MIF; *n* = 12 in MD/VEH; and *n* = 17 in MD/MIF. *p < 0.05, *post hoc* test. ***B***, Comparable results were obtained when the cumulative frequency of mEPSC intervals was analyzed, using KS testing (see main text). ***C***, Averaged data on mEPSCs frequency recorded in dorsomedial striatum neurons of adult male rats (PND90) exposed to MD or control treatment (noMD) at PND3, receiving MIF or VEH between PND26 and PND28. Two-way ANOVA revealed a significant interaction effect of treatment and condition (see main text and Table 4 for statistical details). Group sizes: *n* = 12 cells in noMD/VEH; *n* = 16 in noMD/MIF; *n* = 9 in MD/VEH; and *n* = 15 in MD/MIF. *p < 0.05, *post hoc* test. ***D***, Amplitude of mEPSCs. No condition * treatment effect but a significant main effect of MD and a tendency toward a main effect of MIF treatment was found (see main text and Table 4).

Regarding the mEPSC frequency of CA1 pyramidal neurons, a significant condition × treatment interaction was observed (*F*_(3,59)_ = 6.2, *p* = 0.016; Fig. 4*A*). *Post hoc* analysis showed that the mEPSC frequency was significantly lower in the MD-VEH group than in the control (noMD-VEH) group (*p* = 0.024), whereas other comparisons did not reach significance. Comparison of the distributions of the mEPSC frequency confirmed the difference between the noMD-VEH and MD-VEH group (*p* = 0.003, KS test; Fig. 4*B*). No significant interaction or main effects of condition and treatment were observed regarding the mEPSC amplitude, rise time, or τ of decay (for details, see Table 4).

**Table 4.** Properties of mEPSCs measured in CA1 hippocampal neurons (top) and dorsomedial striatal neurons (bottom) of adult male rats exposed to 24-h MD (or control treatment) at PND3

	noMD VEH	noMD MIF	MD VEH	MD MIF	Condition × treatment	Condition (noMD vs MD)	Treatment (VEH vs MIF)
Hippocampus							
Amplitude (pA)	18.9 ± 0.8	18.1 ± 0.6	18 ± 0.6	18.1 ± 0.7	*F*_(3,61)_ = 0.5; *p* = 0.459	*F*_(3,61)_ = 0.4; *p* = 0.525	*F*_(3,61)_ = 0.2; *p* = 0.634
τ-Rise (ms)	4.2 ± 0.2	3.8 ± 0.2	3.7 ± 0.3	3.9 ± 0.2	*F*_(3,62)_ = 2.3; *p* = 0.132	*F*_(3,62)_ = 0.5; *p* = 0.483	*F*_(3,62)_ = 0.1; *p* = 0.699
τ-Decay (ms)	11.6 ± 0.6	11.4 ± 0.7	11.2 ± 0.7	11.3 ± 0.7	*F*_(3,62)_ = 0.0; *p* = 0.807	*F*_(3,62)_ = 02.; *p* = 0.688	*F*_(3,62)_ = 0.0; *p* = 0.896
Dorsomedial striatum							
Amplitude (pA)	16.1 ± 1.3	16.7 ± 0.7	12.3 ± 0.9	15.7 ± 0.9	*F*_(1,48)_ = 2.0; *p* = 0.161	*F*_(1,48)_ = 5.7; *p* = 0.020	*F*_(1,48)_ = 3.9; *p* = 0.052
τ-Rise (ms)	1.0 ± 0.1	1.0 ± 0.1	0.9 ± 0.1	0.9 ± 0.1	*F*_(1,48)_ = 0.0; *p* = 0.940	*F*_(1,48)_ = 2.2; *p* = 0.139	*F*_(1,48)_ = 0.3; *p* = 0.860
τ-Decay (ms)	6.2 ± 0.5	6.2 ± 0.5	4.7 ± 0.4	5.5 ± 0.5	*F*_(1,48)_ = 0.6; *p* = 0.424	*F*_(1,48)_ = 5.2; *p* = 0.027	*F*_(1,48)_ = 0.5; *p* = 0.483

Half of the animals were treated twice daily with MIF through oral gavage on PND26–PND28.

In the dorsomedial striatum, mEPSC properties observed in control animals were not unlike those recently described for somewhat younger rats (Dorris et al., 2015). A significant condition × treatment interaction (for statistics, see Table 4) was found with respect to the mEPSC frequency (Fig. 4*C*; Table 4). The mEPSC frequency in medium spiny neurons from MD-VEH rats was significantly increased compared to that from noMD-VEH rats (*p* = 0.048). In MIF-treated rats, no such difference was observed. The cumulative frequency distribution of the interevent interval (frequency^−1^) confirms the effects on frequency (KS test with 50-ms bins, difference between cells from noMD-VEH and MD-VEH rats: *p* < 0.0001; difference between cells from MD-VEH and MD-MIF rats: *p* = 0.012; other comparisons: *p* > 0.1; data not shown). With regard to mEPSC amplitude we found a significant main effect of condition (for statistics, see Table 4) and a tendency toward a main effect of treatment, but no interaction (for statistics, see Table 4); in the absence of interaction effects *post hoc* analysis was not performed. In the cumulative frequency distribution of the amplitude, groups did not significantly differ from each other (KS test with 5-pA bins, *p* = 0.071 for noMD-VEH vs MD-VEH; all other comparisons: *p* > 0.1). No significant main or interaction effects were found for rise time. Decay time was significantly decreased after MD but not affected by treatment (Table 4).

Overall, mEPSC frequency in the dorsomedial striatum and CA1 area is consistently increased and decreased respectively by MD; both effects are normalized by MIF treatment. These data parallel the data for the c-Fos staining in these areas and the behavioral performance.

## Discussion

The main finding of this study is that transiently reducing GR activation during a critical developmental period prevents precipitation of cognitive impairments in adulthood associated with early life adversity. These behavioral effects were accompanied by changes in neuronal activity of principal neurons in the dorsal CA1 hippocampus and dorsomedial striatum.

The lower body weight and higher basal CORT level at PND26 following 24-h MD at PND3 support the efficacy of the early life adverse conditions. Basal CORT levels at this age were somewhat higher than expected, considering that plasma was collected standardly before noon. One possibility is that plasma CORT levels already started to rise in rats killed some minutes following removal of the first one. After early life adversity, release of stress mediators and expression of their receptors are sometimes lastingly changed (Liu et al., 1997; Lehmann et al., 2002; Ladd et al., 2004; Oomen et al., 2007; Rice et al., 2008; Xu et al., 2011). Here, we only observed transient changes. The fact that particularly CORT levels and GR expression in the PFC were most clearly changed in the prepubertal period and less so in adulthood seems to justify MIF treatment in this period. Even in the hippocampus, where CORT levels were increased at PND26 after MD, in the absence of changes in GR expression, temporary MIF treatment may help to restore or prevent the development of behavioral deficits. Additionally, it is unclear whether the current ELS treatment affects pubertal timing and may be in fact model dependent. For instance, 3 h of maternal separation from P1 to P14 delayed preputial separation (Bodensteiner et al., 2014) as did immobilizing female rats once per day for 2 h from days 14 to 21 of pregnancy in male offspring (Hernández-Arteaga et al., 2016). However, removing neonatal Sprague Dawley rats from their dams for 6 h daily beginning from P4 to P21 was ineffective (Lau et al., 1996). Clearly, this issue is an interesting question for future research.

The results with the object-in-context task point to a contextual/spatial learning deficit after MD (Kosten et al., 2012; Loi et al., 2017) which was restored by transient MIF treatment. The results from the rIGT task confirm this. Thus, in the maze-based rIGT version used here, learning to discriminate between the advantageous and disadvantageous arm is related to both reward-like and spatial learning, while learning to discriminate the empty arms from the baited arms is just related to spatial learning (van den Bos et al., 2014). Previously, we have shown that reward learning can be disturbed without affecting spatial learning (de Visser et al., 2011b; Koot et al., 2013, 2014). We now show that the reverse is not true: MD affected spatial learning since rats did not discriminate empty from baited arms (despite the presence of cues) and probably as a consequence reward-like learning was compromised. While the deficits in spatial learning were completely restored by MIF, treatment did not completely reverse deficits in reward learning, and seemed to have effects per se (see below). Noteworthy, reward learning was rather weak in control rats, which may be due to mild stress in all groups caused by oral gavage treatment; in agreement, reward learning in the rIGT was shown to be sensitive to early life events (van Hasselt et al., 2012b).

The object-in-context paradigm strongly depends on the dorsal hippocampus (Mumby et al., 2002; Balderas et al., 2008). Learning the maze-based rIGT task depends on the interplay between an emotion-based circuit, involved in assessing the rewarding value of the arms- and a cognitive control/goal-directed circuit, involved in directing long-term instrumental behavior (for review, see de Visser et al.2011a; van den Bos et al., 2014). Recently, a study in mice using the present rIGT suggested a relationship between learning and the hippocampus (Pittaras et al., 2016). Critically, the hippocampus provides the contextual information needed for instrumental learning to be successful, i.e., it affords among other things flexibility in instrumental learning. Instrumental learning in the rIGT depends on the dorsomedial striatum (van den Bos et al., 2014). Indeed the hippocampus (CA1)-dorsomedial striatum connection was shown to be critical in maze-based instrumental learning (Yin and Knowlton, 2006; Devan et al., 2011; Fouquet et al., 2013; Delcasso et al., 2014).

Supporting this and underpinning the behavioral observations, we found, using c-Fos expression and electrophysiology, that MD affected dorsal hippocampus and dorsomedial striatal functioning, which was restored by MIF. Differences between groups were lateralized and particularly strong in the right hemisphere. This agrees with earlier studies, showing, e.g., that chronic stress affects dendritic plasticity in the right PFC, normalizing preexisting differences between hemispheres (Perez-Cruz et al., 2009). Of note, the electrophysiological analysis in our study was not confined to the right hemisphere; hence, the reported average of cells recorded from both hemispheres may underestimate the actual treatment and conditions effects.c-Fos expression following the final rIGT session allowed to assess areas critically involved in the effects of MD and MIF. This global approach pointed, among others, to a key role of the dorsal hippocampus (CA1, CA3) and dorsomedial striatum. We furthermore observed that MD changed c-Fos expression in the right shell of the nucleus accumbens and insular cortex, which was restored by MIF. These changes may also have contributed to the observed behavioral changes and point to a role of MD in reward processing in addition to spatial learning (in line with van Hasselt et al., 2012b). As spatial learning deficits precluded assessing reward learning per se, we cannot draw firm conclusions on this; this would require using reward learning paradigms not containing an element of spatial learning. In a number of areas, MD by itself changed c-Fos expression regardless of MIF treatment, e.g., the left cingulate areas, prelimbic cortex and dentate gyrus, areas of relevance for early life stress (van Hasselt et al., 2012b; Bessa et al., 2013; Taylor et al., 2014; Casement et al., 2015; Chocyk et al., 2015). As the prelimbic cortex is critical in reward learning in the rIGT (de Visser et al., 2011a; van den Bos et al., 2014), this may have contributed to the behavioral deficits seen in the MD-VEH group (not sensitive to MIF). Clearly, this requires more research.

While c-Fos immunoreactivity can identify global changes in neuronal activity, upregulation (dorsomedial striatum) and downregulation (CA1) of expression is difficult to relate to functional changes in neuronal activity. Contextual learning and memory critically depend on hippocampal glutamatergic transmission (Mitsushima et al., 2011; Middei et al., 2013), which is affected by early life conditions and CORT (Zhang et al., 2010; Bagot et al., 2012a,b; for review, see Krugers et al., 2010; Joëls et al., 2012; Popoli et al., 2012;). Therefore, we also recorded spontaneous glutamatergic transmission in the two key areas. After MD, mEPSC frequency in CA1 neurons from VEH-treated rats was reduced, but not in MIF-treated rats. Yet, spontaneous glutamatergic transmission in the dorsomedial striatum was increased after MD and restored by MIF. Since medium spiny neurons are GABAergic, this may indicate lower functional output of this area. Of note, electrophysiological properties may differ between dopamine-1 and dopamine-2 receptor-expressing cells in the striatum, but this does not seem to be the case for mEPSC frequency (Ma et al., 2012); we therefore did not attempt to distinguish between these cell populations in our recordings. Collectively, these data suggest that early life stress targets glutamatergic transmission, an effect that can be restored by temporary MIF treatment. However, since behavioral and electrophysiological measurements were only performed in adult animals, the order (and possible causality) of changes cannot be inferred from the present study. Additionally, a limitation of the design is that the number of offspring from each litter was not statistically corrected warranting careful interpretation. To maintain practical feasibility, we chose an alternative approach in which every outcome was based on animals from at least four different litters and every experiment was randomized and performed in a double-blind manner, from treatment to analysis.

Brief treatment with MIF was shown to normalize changes in rat hippocampal structural and functional plasticity resulting from chronic stress after just 3 d of treatment (Krugers et al., 2006; Oomen et al., 2007) and even after a single day of MIF administration (Hu et al., 2012). Also, juvenile stress on P26-P28 was shown to sensitize rats to (renewed) stress in adulthood (Tsoory and Richter-Levin, 2006). These are the main reasons why we opted for a brief treatment in the prepubertal period here as well. Moreover, by only treating rats during a brief period, effects of sustained treatment into adulthood did not confound our findings. Interestingly, we observed long-lasting effects after the brief prepubertal treatment, which suggests involvement of epigenetic pathways (Hornung and Heim 2014). Clearly, the mechanism by which the MIF effects are established requires extensive follow-up investigations. To what extent our observations hold true for other models of early life adversity also awaits further investigation, yet the fact that normalization of contextual memory by brief prepubertal MIF treatment was also seen in the limited bedding and nesting model of early adversity in mice (Arp et al., 2016) supports a more general efficacy of this paradigm. Importantly, MIF was administered after early life adversity was terminated, which is interesting from a translational point of view. The current results provide a promising basis for new intervention strategies in humans exposed to early life adversity. Future studies will be needed to determine the minimal duration and window of opportunity for effective treatment window after early life adversusersity.

MIF discriminates well between GR and mineralocorticoid receptors (MRs; Moguilewsky and Philibert, 1984; Flores et al., 2006; Johanssen and Allolio, 2007). However, acute MIF treatment may be associated with brief increases in plasma CORT levels (Nedvídková et al., 1997; Zalachoras et al 2014), which could increase MR activation while GR is blocked, shifting the MR/GR activation ratio toward MR. Moreover, MIF also binds with high affinity to progesterone receptors (Gallagher and Young, 2006). We can therefore not exclude that the normalizing effects partly involve progesterone receptors or a shift in MR/GR ratio. The present findings form the basis for future studies with more selective GR antagonists that pass the blood-brain barrier.

In conclusion, transient prepubertal treatment with MIF fully normalizes hippocampus-striatum-dependent contextual memory/spatial learning deficits in male rats exposed to early life adversity, likely involving glutamatergic transmission.

## Synthesis

Reviewing Editor: Liisa Galea, University of British Columbia Decisions are customarily a result of the Reviewing Editor and the peer reviewers coming together and discussing their recommendations until a consensus is reached. When revisions are invited, a fact-based synthesis statement explaining their decision and outlining what is needed to prepare a revision will be listed below. The following reviewer(s) agreed to reveal their identity: Kevin Bath.

After consultation with the reviewer and reviewing editor, some minor revisions are required mostly concerning the methods, with some statistics and the justification of the timing of mifepristone relative to puberty.

1) The authors gave mifepristone on P26-P28 but call it pubertal treatment but puberty in male Wistar rats appears to be around P40. The language, including that in the title, should be changed to “pre-puberty” not early puberty.

2. Why were these days (P26-28) chosen - when was the peri-pubertal activation seen in Tzanoulinou et al 2014 paper?

3)What was the number of offspring from each litter that was used? If more than 1 or 2 this needs to be accounted for statistically using litter effects.

4) Line 138 - were the rats triple or pair housed? If both was there a difference in outcome based on pair vs triple housing?

5) Lux seems very low for EPM testing. Could they reference these low levels?

6)How many sections were analysed for c-fos per area?

7)LSD is a very liberal test - consider a different test- but also within the results section planned comparisons are referred to - while LSD and Planned comparisons have the same formula it is an important distinction for the reader and generally planned comparisons should be subjected to a Bonferroni to control for type 1 error correction. (lines 447, 417)

8) Did the treatments affect pubertal timing?

Minor

Line 255 - inedible. Uneatable

May want to indicate the weaver study was also in males.

Line 458 - 'little' learning or impaired learning?

Figure 3- symbols appear to be different sizes

Line 370-372 - p values?
